# Alterations of Regional Homogeneity in Parkinson’s Disease: A Resting-State Functional Magnetic Resonance Imaging (fMRI) Study

**DOI:** 10.7759/cureus.26797

**Published:** 2022-07-12

**Authors:** Hong Zhu, Haokai Zhu, Xiaozheng Liu, Yingcan Zhou, Shuangshuang Wu, Fuquan Wei, Zhongwei Guo

**Affiliations:** 1 Department of Geriatrics, Tongde Hospital of Zhejiang Province, Hangzhou, CHN; 2 Medicine, Zhejiang Chinese Medicine University, Hangzhou, CHN; 3 Department of Radiology, The Second Affiliated Hospital and Yuying Children’s Hospital, Wenzhou, CHN; 4 Department of Radiology, Tongde Hospital of Zhejiang Province, Hangzhou, CHN

**Keywords:** local brain activity, frequency bands, regional homogeneity, resting-state functional magnetic resonance imaging, parkinson disease

## Abstract

Objective

The objective of this study is to investigate the regional homogeneity (ReHo) of spontaneous brain activities in Parkinson’s disease (PD) patients.

Methods

In total, 20 PD patients and 20 matched normal controls (NCs) participants were recruited for this study. The regional homogeneity (ReHo) approach based on resting-state functional magnetic resonance imaging on a 3T MRI system was used to investigate local brain activity. We examined activity in two frequency bands, slow‐4 (0.027-0.073 Hz) and slow‐5 (0.010-0.027 Hz). Two-sample t-tests were used to determine the between-group differences in the ReHo data. Pearson correlation analysis was used to explore the relationships between the ReHo values and clinical indices in PD patients.

Results

Compared with NCs, PD patients showed decreased ReHo values in the right middle occipital gyrus, right cuneus, and left superior occipital gyrus, and increased ReHo values in the right middle frontal gyrus in slow‐4. PD patients showed decreased ReHo values in the right calcarine, left calcarine, and right precentral gyrus compared with NCs in slow‐5. Correlation analysis showed that disease duration was negatively correlated with ReHo values in the right precentral gyrus in PD patients.

Conclusions

These results indicate that several brain regions were altered in PD patients. The regions are associated with the visual network-related cortex, motor cortex, and default mode network. The findings provide new insights into the neuropathophysiology of PD.

## Introduction

Parkinson’s disease (PD) is a common neurodegenerative disease in the elderly and has typical clinical manifestations, including resting tremors, muscle rigidity, bradykinesia, and postural gait disorder [1]. In addition, non-motor symptoms, such as olfactory decline, sleep disorders, anxiety, depression, and cognitive impairment, are associated with PD. Non-motor symptoms and motor complications are responsible for most of the disability burden [2]. These symptoms not only bring pain to patients, but they also impose a heavy burden on families and society. Understanding the pathogenesis of PD will be helpful for discovering effective therapies and early interventions.

In recent decades, an increasing number of neuroimaging studies have explored the pathophysiology of PD by using different imaging modalities such as diffusion tensor imaging [3], positron emission tomography (PET) [4,5], single‐photon emission computed tomography (SPECT) [6], and functional magnetic resonance imaging (fMRI) [7,8]. Resting-state fMRI (rs-fMRI) is a non-traumatic brain imaging technique that measures blood oxygen level dependency (BOLD) [9]. Compared with PET or SPECT, rs‐fMRI is simpler, as it does not require radiotracers and is more widely available. Regional homogeneity (ReHo) is a common, reliable, and efficient method to investigate resting-state regional brain activity in neuropsychiatric disorders, including PD [8,10-12]. The consistency of neuronal activity is determined by evaluating the similarity of blood oxygenation level-dependent (BOLD) signals of adjacent voxels in a certain time series [13]. Increases or decreases in ReHo reflect hyperactivity or hypoactivity of spontaneous activity in local neurons and the changes in brain activity homogeneity [14]. Many studies have used ReHo to research alterations of local brain activity. Li et al. [15] reported significantly decreased ReHo in 23 PD patients in the right precentral gyrus and right postcentral gyrus compared with 20 NCs. However, Jiang et al. [16] reported that PD patients with postural instability and gait difficulty exhibited significantly increased ReHo in the right precentral gyrus (M1) and right postcentral gyrus (S1) compared with NCs. The heterogeneous findings of these two previous studies may result from the absence of frequency division. 

Abnormal brain function is associated with specific frequency bands [17]. The analysis of frequency bands of rs-fMRI signals may provide an effective way to study PD pathology and the physiological mechanism of the dopamine pathway in the brain [17]. In a previous study, the frequency spectrum was decomposed into five different frequency bands, including slow-6 (0-0.01 Hz), slow-5 (0.01-0.027 Hz), slow-4 (0.027-0.073 Hz), slow-3 (0.073-0.198 Hz), and slow-2 (0.198-0.25 Hz). Slow-6, slow-3, and slow-2 oscillations are discarded because they mainly reflect very low-frequency drift, white matter signals, and high-frequency physiological noise, whereas slow-4 and slow-5 oscillations primarily correlate with gray matter and are beneficial for identifying correlations between functional processing and diseases [18,19]. Hou et al. [20] examined activity in two frequency bands, slow-4 and slow-5, in 109 PD patients and reported that decreased amplitude of low-frequency fluctuation (ALFF) in the striatum and increased ALFF in the midbrain compared with healthy control subjects were more significant in slow‐4.

In this study, we utilized rs‐fMRI and ReHo to investigate alterations of local brain activity in different frequency bands in PD patients. These findings will provide further insight into the neurophysiological mechanisms of PD.

## Materials and methods

Participants

The study involved 20 PD patients and 20 sex, age, and education level-matched normal controls (NCs). All PD patients were diagnosed according to the clinical criteria of the Movement Disorder Society [21]. The exclusion criteria for patients were: 1) magnetic resonance (MR) contraindications; 2) history of epileptic attack, syncope attack, head trauma, drug abuse, alcoholism, or psychiatric diagnosis; 3) patients with cerebral parenchymal lesions; and 4) presence of moderate to severe head tremor that may potentially interfere with the fMRI scan. NCs who had no history of neurologic or psychiatric diseases were enrolled. Demographic characteristics, including sex, age, education level, and disease duration, were collected from all participants. All PD patients underwent a series of detailed neurological examinations by professional neurologists. Patients were evaluated using the motor part of the Unified Parkinson’s Disease Rating Scale (UPDRS-III) and the Hoehn-Yahr stage (HY) while off medications. Mini-Mental State Examination (MMSE) was used to assess overall cognitive function. All scales were executed by trained clinical neuropsychologists. The research ethics committee approved the study, and each participant provided written informed consent before MR scanning.

Data acquisition

All MR imaging examinations were performed with a 3.0 T MR system (3.0 T Discovery MR750, GE Healthcare, Chicago, Illinois) and an eight-channel head coil. T1-weighted high-resolution anatomical images were acquired using a spoiled gradient refocused acquisition sequence: repetition time = 7.68 ms, echo time = 3.43 ms, flip angle = 12°, field of view = 256 mm2, slice thickness = 1 mm, layer spacing = 1 mm, and voxel size = 1 mm × 1 mm × 1 mm. Rs-fMRI data were collected transversely with an echo-planar imaging sequence using the following settings: repetition time (TR) = 2000 ms, echo time (TE) = 30 ms, flip angle = 90°, field of view (FOV) = 220 mm × 220 mm, slices = 36, in-plane matrix = 64 × 64, thickness = 3 mm, layer spacing = 1 mm, and voxel size = 3.44 mm × 3.44 mm × 4 mm. One hundred eighty volumes were acquired from each participant, which resulted in a total scan time of 360 s.

 Data processing

Data of each fMRI series contained 240 time points. The first 10 time points were discarded because of the instability of the initial MRI signal and the adaptation of the participants to the environment. Preprocessing of remaining fMRI data was conducted using Data Processing Assistant for Resting-State fMRI V2.1. Time-series data were first slice-time corrected and aligned to the first image of each session for motion correction. Each participant’s head motion parameters were examined. Functional images of each participant were coregistered to high-resolution anatomical images and underwent spatial normalization into the standard Montreal Neurological Institute brain space. All images were then resampled into voxels that were 3 × 3 × 3 mm3 in size. Then, linear drift was removed. To examine the frequency effects on brain activity changes in patients, temporal band-filtering was further performed in the slow-5 (0.01-0.027 Hz) and slow-4 (0.027-0.073 Hz) bands.

For a given voxel, Kendall’s coefficient of concordance (KCC) was computed on the ranked BOLD time series within a cluster formed by the given voxel and its nearest neighbors (27 voxels in the present study). The resultant KCC was the ReHo for the voxel. To reduce the influence of individual variation in KCC values, ReHo map normalization was performed by dividing the KCC of each voxel by the averaged whole-brain KCC. Global-mean normalized ReHo maps were then spatially smoothed using an isotropic Gaussian kernel with a full width at half maximum of 6 mm. 

Statistical analysis

For comparisons of demographic and psychometrics, we used a two-sample t-test when samples had a standard normal distribution and the Mann-Whitney U test when samples exhibited a skewed distribution, while a Chi-square test was applied to compare sex distributions.

Two-sample t-tests were used to determine the between-group differences in the ReHo data. Age, sex, and frame-wise displacement of head motion were treated as non-interest covariates for between-group comparisons. AlphaSim (AlphaSim, Tauranga, New Zealand), a program based on Monte Carlo simulation and implemented in AFNI, was used for multiple comparison correction. All of the results are presented at a statistical threshold of p<0.05 (corrected) by combining a height threshold (p<0.005) and cluster size greater than 25 voxels within the intersection mask. Pearson correlation (statistical significance level p<0.05) was used to quantify the correlation between ReHo and clinical data.

## Results

Demographic and clinical data

There were no significant differences in sex, age, years of education, or handedness between PD patients and NCs (Table 1).

**Table 1:** Demographic and clinical data Data represented as mean ± SD. Data were analyzed using independent-samples t-tests. PD - Parkinson’s disease; NC - normal controls; M - male; F - female; UPDRS-III - Unified Parkinson’s Disease Rating Scale (part three); HY - Hoehn and Yahr stage; MMSE - Mini-Mental State Examination

	PD (n=20)	NC (n=20)	t/χ^2^	p
Sex (M/F)	20 (13/7)	20 (10/10)	0.921	0.337
Age (years)	67.4±7.4	65.4±4.4	-1.042	0.305
Education (years)	11.6±2.4	12.1±2.5	-0.645	0.523
Handedness (R/L)	20:0	20:0	-	-
Duration (years)	3.9±2.9	-	-	-
UPDRS-III	34.3±9.3	-	-	-
HY	3.1±0.9	-	-	-
MMSE	26.2±3.5	27.8±1.4	1.951	0.062

ReHo analysis

**Figure 1:**
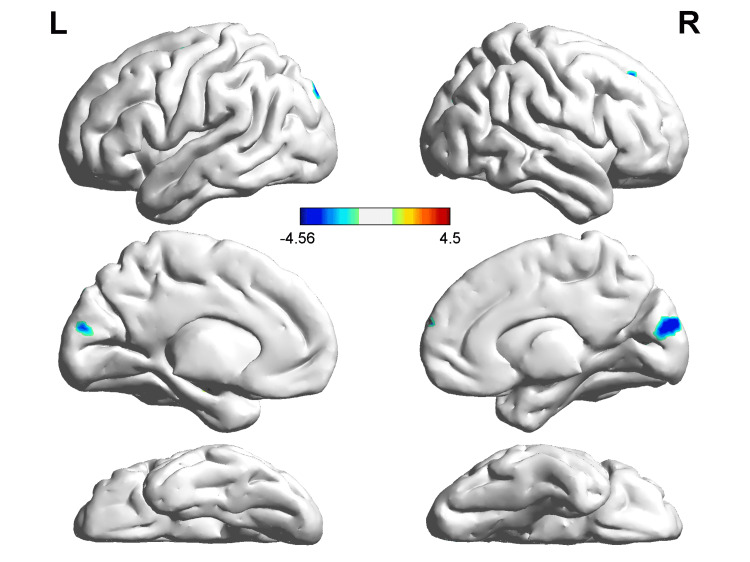
ReHo alterations in PD patients compared with NCs in slow‐4 The voxels with warm colors represent PD-related ReHo increases, and cool colors indicate decreases. PD - Parkinson’s disease; NC - normal controls; ReHo - regional homogeneity

**Figure 2:**
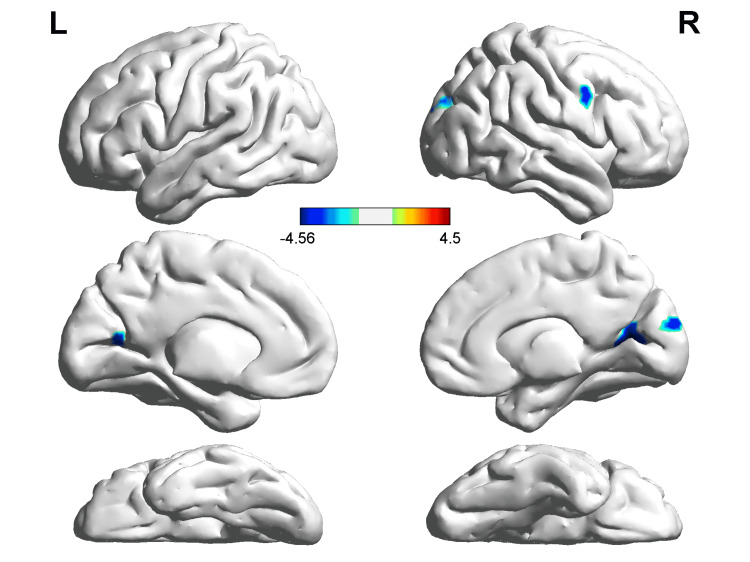
ReHo alterations in PD patients compared with NCs in slow‐5 The voxels with warm colors represent PD-related ReHo increases, and cool colors indicate decreases. PD - Parkinson’s disease; NC - normal controls; ReHo - regional homogeneity

In comparison with NCs, PD patients showed decreased ReHo values in the right middle occipital gyrus (MOG), right cuneus, and left superior occipital gyrus (SOG), and increased ReHo values in the right middle frontal gyrus (MFG) in slow‐4. PD patients showed decreased ReHo values in the right calcarine, left calcarine, and right precentral gyrus compared with NCs in slow‐5 (Table 2, Figures 1, 2).

**Table 2:** Brain regions with significant differences in the PD group compared with the NC group PD - Parkinson’s disease; NC - normal controls; MNI - Montreal Neurological Institute; BA - Brodmann area

Brain regions	Voxels	MNI coordinates			BA	T-value
		x	y	z		
Slow‐4						
Right middle occipital gyrus	32	27	-81	12	18	-4.5608
Right cuneus	89	6	-93	21	18	-4.1234
Left superior occipital gyrus	26	-15	-87	39	19	-4.0024
Right middle frontal gyrus	98	18	-6	45	-	4.1546
Slow‐5						
Right calcarine	250	18	-57	9	17	-4.8847
Left calcarine	36	-15	-63	12	17	-3.9341
Right precentral gyrus	31	60	6	39	6	-4.1004

**Figure 3:**
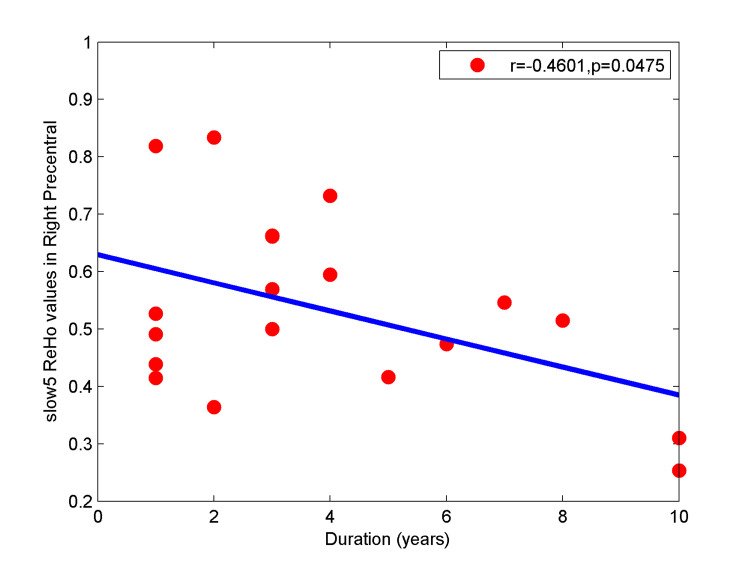
Pearson correlation analysis between the ReHo values in the right precentral gyrus and duration ReHo - regional homogeneity

Correlation analysis showed that disease duration was negatively correlated with ReHo values in the right precentral gyrus in PD patients (Figure 3, r = −0.4601, p<0.05).

## Discussion

In the present study, we examined alterations of local brain activity in PD patients and NCs. Compared with NCs, PD patients showed decreased ReHo values in the right MOG, right cuneus, left SOG, right calcarine, left calcarine, and right precentral gyrus, and increased ReHo values in the right MFG.

First, the findings indicate that the PD group had reduced consistency of functional activity in the visual network-related cortex, specifically the bilateral calcarine, right MOG, left SOG, and right cuneus, which suggests impaired visual information integration. Multiple studies of visual function in PD have shown that PD can present with impaired visual collection and visual space flip abilities, reduced spatial perception, and visual cortex loss in the early stage, and can manifest visual hallucinations in the later stage. Therefore, the findings of this study are consistent with previous research reports [22,23]. Tessitore et al. [24] reported that PD patients with freezing of gait had impaired implementation attention networks and visual networks, which is consistent with our results. Walking and maintaining posture is not a completely automated process but requires the local integration of external information [25,26]. It has been shown that severe visual impairment that impacts motor, cognitive and other functions is present in nondementia PD patients [22]. Additionally, a prior imaging study showed that local cerebral blood flow in visual-related regions is reduced in PD patients compared with controls [27].

Secondly, our results showed decreased ReHo values in the right precentral gyrus in PD compared with NC. This brain region is part of the motor cortex and is involved in the striatal-thalamic-cortical circuit [28]. Automaticity of gait and motor planning involves precise control of a series of complex actions and adjustments in response to feedback from multiple sources of information. This process uses not only motor cortex outputs and inputs, but also information from visual, auditory, proprioceptive, cognitive, and other systems to form a complete sensorimotor integration process. Previous studies have reported both structural and functional motor cortex damage in PD [29,30]. Functional lesions of the motor cortices play an important role in PD neuropathophysiology [31]. Dopamine insufficiency in the nigrostriatal pathway may cause decreased ReHo values in the motor cortex [31]. Decreased ReHo in this region of Parkinson's patients has been reported in several studies [32,33]. A meta-analysis of ReHo in PD reported that ReHo is decreased in the right precentral gyrus (premotor cortex extending to the primary motor cortex, M1) in patients compared with normal controls [34]. In addition, Li Y et al. [15] reported that ReHo was significantly decreased in PD patients compared with controls in multiple motor network areas, including the right precentral gyrus. Li J et al. [35] reported that PD patients with fatigue had decreased ReHo in the right precentral gyrus compared with NCs, which is consistent with our findings. Within the PD group, we found that disease duration was negatively correlated with ReHo values in the right precentral gyrus, which is consistent with clinical characteristics in PD that dopamine deficiency worsens as the disease progresses.

Thirdly, our study showed increased ReHo values in the right MFG in patients with PD compared with NCs. The right MFG is a component of the default mode network (DMN), which is most active at rest [36]. The DMN is related to situational memory, self-consciousness, sustained cognition, and the detection of the brain's external environment [37,38]. Previous studies have indicated that the default network has varying degrees of impairment in PD, which may be associated with the reduction of dopamine neurotransmitters [39,40]. A previous study suggested that the enhancement of frontal lobe functional activity is a compensatory or antagonistic mechanism in PD; with increased motor symptoms, the frontal lobe restrains motor symptoms by increasing activity in an individual with PD [41].

Several limitations should be noted in the present study. First, the present study is a cross-sectional design. Longitudinal studies that dynamically evaluate changes in brain function during the duration of the disease are needed. Second, the small sample size may cause statistical errors. To increase the statistical efficiency and sensitivity, studies with large sample sizes are necessary. Third, because of different heartbeats and respiratory frequencies of participants during data acquisition, low-frequency filtering and delinear drift may not completely remove the interference of physiological noise caused by heartbeat and respiratory frequency on BOLD signals. Fourth, ReHo is a reliable and efficient method to measure resting-state regional brain activity, but explaining the potential neurobiological mechanisms of ReHo has been a major challenge [42].

## Conclusions

In the present study, we used rs-fMRI and ReHo to examine local brain function in PD and found that multiple brain regions were altered in PD. The altered regions are associated with the visual network-related cortex, motor cortex, and DMN. These findings suggest that these regions may play important roles in the neuropathophysiology of PD.
